# The potential for the double risk of rabies and antimicrobial resistance in a high rabies endemic setting: detection of antibiotic resistance in bacterial isolates from infected dog bite wounds in Uganda

**DOI:** 10.1186/s13756-022-01181-0

**Published:** 2022-11-13

**Authors:** Stevens Kisaka, Fredrick E. Makumbi, Samuel Majalija, Moses Muwanga, S. M. Thumbi

**Affiliations:** 1 University of Nairobi Institute of Tropical and Infectious Diseases, Nairobi, Kenya; 2 School of Public Health, Makerere University, Kampala, Uganda; 3 College of Veterinary Medicine, Animal Resources and Biosecurity, Makerere University, Kampala, Uganda; 4 Department of Medicine, Entebbe General Referral Hospital, Entebbe, Uganda; 5 Rabies Free Africa, Washington State University, Pullman, USA; 6 Paul G Allen School for Global Animal Health, Washington State University, Pullman, USA

**Keywords:** Post-expsoure treatment, Dog bite wound, Wound infection, Rabies, Antimicrobials, Antimicrobial resistance

## Abstract

**Background:**

Post-exposure treatment for dog bites in humans aims at alleviating the risk of rabies and promoting wound healing. Wound healing may be complicated by bacteria. This study identified the different bacteria and their antibiotic susceptibilities in infected dog bite wounds (DBWs) in Uganda.

**Methods:**

A cross-sectional study was conducted among 376 dog bite patients. Wound swabs from patients with infected DBWs were collected and inoculated into recommended media. They were cultured for both aerobic and anaerobic bacteria. All isolated bacteria were identified based on colony characteristics, gram stain, and standard biochemical tests. Molecular identification was performed for strains that were resistant to three or more antibiotics. Antibiotic susceptibility testing was conducted using the disc diffusion method following the modified Kirby-Bauer method. The data were analysed using Stata version 15 software.

**Results:**

Approximately half of the patients (52.9%, 199/376) presented with infected wounds. Majority of the swabs (84.4%, 168/199) were culture positive, and yielded a total of 768 isolates where about half (52.9%, 406/768) were gram positive bacteria, and about two-thirds (64.6%, 496/768) were recovered from category II wounds. Among the gram positive bacteria, 339 (83.5%) were aerobes where *Staphylococcus aureus* (103, 30.4%), Coagulase-negative staphylococci (68, 20.1%), and *Corynebacterium spp *(33, 9.7%) had the highest prevalence. For the 362 Gram negative isolates, 217 (59.9%) were aerobes and the commonest isolates were *P. maltocida* (64, 29.5%), *Capnocytophaga canimorsus* (36, 16.6%) and *P. canis* (26, 12.0%). Gram-positive isolates were resistant to metronidazole (93.6%), oxacillin (68.5%), ceftriaxone (14.6%) and amoxicillin/clavulanic acid (14.0%). Gram negative isolates were resistant to metronidazole (100%), ampicillin (30.7%), oxacillin (29.3%), and doxycycline (22.9%). Multidrug resistance was in 105 (29.0%) and 121/406 (29.8%) of the gram-negative and gram-positive isolates, respectively. All gram-positive isolates were susceptible to vancomycin and ciprofloxacin.

**Conclusions:**

Infection rates of DBWs in Uganda are high and the dominant bacterial isolates are *Staphylococcus aureus, Pasteurella spps*, and *Capnocytophaga canimorsus.* Multidrug resistance to commonly used antibiotics is high. The recommendation in the Uganda Clinical Guidelines to use metronidazole in the management of DBWs should be reviewed. DBWs should be enlisted for routine antimicrobial resistance surveillance and rational use of antimicrobial agents should be promoted.

**Supplementary Information:**

The online version contains supplementary material available at 10.1186/s13756-022-01181-0.

## Background

Dog bite injuries among people are on the rise and constitute a huge health burden to societies globally. In the United States, there are approximately 4.5 million dog bite injuries annually [1] while in the United Kingdom, the 7227 hospital admissions for dog bites between March 2014 and February 2015, indicated a 76% increase compared to the past decade [2]. In Uganda, it is estimated that there are over 30,000 animal bites reported to healthcare facilities annually despite ongoing interventions like health education [3]. The country, with a 10% rabies vaccination coverage for dogs, had approximately 486 suspected human rabies deaths between 2001 and 2015 [4, 5]. It is also estimated that the country would experience approximately 592 deaths due to rabies in the absence of PET [6]. In such low-income countries with endemic dog-transmitted human rabies, pre-exposure prophylaxis (PrEP) with rabies vaccine is almost unavailable and dog vaccination coverage is low [7, 8]. This means that dog bite injuries carry with them a high risk for rabies.

Management of dog bite wounds (DBWs) mainly aims at prevention of rabies, tetanus, and wound infection. In terms of infection, DBWs present with complex bacteriology, which may influence treatment outcomes like wound infection [9]. However, the bioburden of dog bites varies in terms of sources, species, and quantity of bacteria. It is estimated that, on average, an infected dog bite wound contains 2–5 different species of bacteria depending on whether it is an abscess, purulent or non-purulent. Additionally, the source of bacteria is also complex in that they are usually derived from either the oral flora of the dog, the skin of the victim, the environment, or all three [10, 11]. Therefore, DBWs are usually contaminated and / or infected by a wide variety of microorganisms from diverse sources.

DBWs usually present with mixed anaerobic and aerobic bacteria. Among the aerobes isolated are *Staphylococcus ssp, Pasteurella spp. (P. multocida, P. canis, P. dagmatis, Capnocytophaga canimorsus, Bacillus, Actinomyces and Corynebacterium spps* and many others. However, in all studies, *Pasteurella spp* is the most common comprising up to 50% of isolates from dog bites [12, 13] because these organisms are normal flora in the oral cavity of dogs. In addition, an emerging syndrome of methicillin-resistant *Staphylococcus aureus* (MRSA) infections shared between pets and people has been described [14]. Still, anaerobes are isolated more frequently from abscesses than other types of infections [10]. These include, but are not limited to, *Bacteroides spp, Clostridium, Fusobacterium, Porphyromonas, Prevotella, Propionibacterium, Bacteroides,* and *Peptostreptococcus* [15].

This complexity of microbial wound infection propels the need for microbiological examination to occupy a critical step in the treatment of DBWs as it guides the choice of antibiotics. On the other hand, the use of prophylactic antibiotics in managing DBWs has raised considerable controversy. When a meta-analysis of eight randomized trials was conducted, there was a benefit with antibiotic prophylaxis in animal bites compared with untreated patients [16]. Similarly, there were differences in bite wounds to the hand where the infection rate dropped from 28 to 2% with the use of prophylactic antibiotics [17]. In contrast, a review of nine trials yielded no differences in rates of infection between those who had received prophylactic antibiotics and those who had not. Nonetheless, there is consensus supporting the use of antibiotic prophylaxis for high-risk bite wounds [18].

Much as the usefulness of bacterial assessment is still questionable and controversial [19–21], it forms the basis of sensitivity tests that are recommended in Uganda’s National Clinical Guidelines (UCG). The guidelines further restrict antimicrobial administration to bite wounds with a high risk of infection [22]. However, microbiological examination is not routinely done in the post-exposure treatment (PET) centers in Uganda, a high rabies endemic country. Consequently, the presence of bacterial strains, especially of greater public health significance, commonly present in cases of DBWs remains obscure. Besides the economic consequences and eventual side effects, indiscriminate antibiotic therapy presents a risk of increasing antimicrobial resistance in various bacterial strains [9]. Worse still, their sensitivity to recommended antibiotics cannot be predicted yet antimicrobial resistance in strains isolated from DBWs has been reported elsewhere [15, 23]. Therefore, this study not only examined the bacteriology of DBWs but also evaluated the sensitivity of isolates to antibiotics recommended in UCG, in addition to those commonly used to treat wounds.

## Methods

### Study design and area

A cross sectional study design with a quantitative approach was used. The study was in Uganda, a country with a 10% rabies vaccination coverage for dogs and an average of 14,865 dog bites and 36 human rabies deaths annually [5, 24]. The study sites were two referral healthcare facilities, namely Mulago National Referral Hospital (Kampala Capital City) and Entebbe General Referral Hospital (Wakiso district). These referral facilities were purposely selected to represent healthcare facilities providing dog bite post-exposure treatment (PET) in the two rabies endemic districts. In addition, they provide PET to most of the dog bite patients in the two districts. In Wakiso district, there are 64,940 dogs, with approximately 13.5% of households owning an average of 1.7 dogs per household. Conversely, there are 58,100 dogs in Kampala city, with about 7.7% of households owning an average number of 1.9 dogs per household [25].

### Study population and data collection

Between April and October 2019, all patients presenting with dog bites at the two health facilities for first-time PET were consented and recruited into the study. All new patients were enrolled consecutively. Patients with category I bites (44/420, 10.5%) who were assessed as not requiring PET were excluded, leaving a total of 376 study participants. The health-seeking behaviour of the 376 participants and their exposure to the risk of rabies has been decribed in a previous paper [26]. Quantitative data were collected using questionnaires to record socio-demographic and other patient-related factors (Additional file 1). All data collection tools were in English and Luganda languages and had been pretested on animal bite patients in Mukono Health Center IV, in Mukono district, Uganda.

The World Health Organization (WHO) classified DBWs into three categories: category I (victim's skin is intact), category II (minor scratches without bleeding from contact, or licks on broken skin), and category III (one or more bites, scratches, licks on broken skin, or other contact that breaks the skin). This study followed this classification. DBWs were further described in terms of anatomic location (lower limb, upper limb, torso, head/face and combination of these) and the presence of clinical signs of infection and severity. Infection of the DBW was determined by the clinicians based on the existence of one major sign (pus, fever, or leukocytosis) or, at least three minor signs: mal-odour erythema, oedema, subcutaneous emphysema, and tissue necrosis as earlier described [9]. Only infected wounds were subjected to bacteriological analysis.

### Sample collection, culturing and identification

The wound was cleaned with normal saline. A sterile moistened cotton swab was used to obtain a sample of pus or wound secretion, purulent exudates, or wound discharge from each study participant. To avoid contaminating the swab with commensal bacteria from the skin surrounding the wound, the area around the wound was first cleaned with cotton and normal saline. In addition, care was taken to restrict the swab to the wound while avoiding contact with the intact skin. The swab was then immersed in a container of Brain Heart Infusion (BHI) transport medium. For abscesses and puncture wounds, the specimens for bacteriologic examination were obtained by needle aspiration and mini-swabs, respectively. The samples collected each day were transported to the microbiology laboratory at Makerere University at the College of Veterinary Medicine, Animal Resources and Biosecurity (MakCoVAB).

In the laboratory, the swab samples were inoculated onto MacConkey agar, mannitol salt agar, pseudomonas agar media, blood agar plate, and chocolate agar plate (Oxoid, Ltd.). Those inoculated on the previous three media were incubated for 18–24 h at 37 °C. The samples on BAP and CAP were incubated in a humid, 5% carbon dioxide environment for 18–22 h at 35 °C–37 °C. The plates that were aerobically incubated were examined for bacterial growth after the standard incubation timelines. For those that showed growth, they were further sub-cultured on their respective media to obtain pure cultures. However, if any plate did not show growth after this time, it was incubated for a further 24 h. Upon obtaining pure colonies, they were subjected to Gram stain, colony morphology, and biochemical tests (Oxoid, Ltd.). Species identification of the isolates was performed from pure colonies using classical biochemical tests according to the standard guidelines [27]. For isolates that were multidrug resistant, DNA was extracted from their suspensions using the QIAamp DNA mini kit (QIAGEN) following the manufacturer’s protocol with minor modifications. DNA was eluted in 50 ul of TE buffer. DNA quantification and quality control were done using the NanoDrop 2000c (Thermoscientific) following the manufacturer’s protocol. Specific primer sets for each isolate were used in PCR. PCR amplifications were performed using 10ul of the eluted DNA in a 50-μl mixture containing 250 μM each deoxynucleoside triphosphate (Life Technologies), 1.5 U of Taq DNA polymerase (Life Technologies), 20 mM Tris–HCl (pH 8.4), 50 mM KCl, and 2 mM MgCl2. The PCR tests were run in a programmable thermal cycler (BioRad.) Amplification conditions consisted of 10 min at 95 °C, followed by 40 cycles of 1 min at 95 °C, 30 s at 55 °C, and 30 s at 72 °C, with a final step of 5 min at 72 °C. The success of the amplifification was determined by ethidium bromide staining following the resolution of products by 1.5% agarose gel electrophoresis. Each experiment included sterile water as a negative control and a positive control.

### Antimicrobial susceptibility testing

The disc diffusion method was used to conduct antimicrobial susceptibility testing on each of the identified organism. It was carried on Muller Hinton agar (MHA) and blood agar as stated in the guidelines of the Clinical and Laboratory Standards Institute. Furthermore, the zones of inhibition were measured, read and interpreted in line with CLSI [28]. Antimicrobials that were recommended in the UCG to manage DBWs were given priority at testing i.e. metronidazole, methicillin, amoxicillin/clavulanic acid, doxycyline and cotrimoxazole (trimethoprim-sulfamethoxazole [22]. In addition, common antibiotics used in routine clinical practice were also tested, including: streptomycin (10 μg), vancomycin (30 μg), oxacillin (5 μg), gentamicin (10 μg), ciprofloxacin (5 μg), ceftriaxone (30 μg), chloramphenicol (30 μg), ampicillin (10 μg), and imipenem (10 μg). A strain that was not susceptible to at least one antimicrobial in three or more antimicrobial classes was taken to be multidrug resistant (MDR) as earlier defined [29].

### Quality assurance and control

The questionnaire, which was used to collect data from participants, was pretested, and quality control measures were taken in all laboratory procedures. We used control strains, including both susceptible and resistant strains, which served to monitor test performance. *Staphylococcus aureus* ATCC 25923 and *Escherichia coli* ATCC 25922 strains were used as controls while performing susceptibility tests for gram positive and gram negative bacteria, respectively. These were obtained from the National Collection of Type Cultures (UK) through the Microbiology Laboratory at MakCoVAB.

### Data management and analysis

“Antimicrobial Susceptibility” was used to describe the susceptibility of bacteria to antibiotics and it was recorded and categorized as “Susceptible, Intermediate, or Resistant” based on the break-point readings. At univariate analysis, descriptive statistics that included mean (± standard deviation) for continuous variables like age were obtained, while for categorical variables such as Strain and Gram positivity/negativity, frequencies and proportions (percentages) were generated. Proportions (percentages) were used to describe the antimicrobial susceptibility for each of the bacterial isolates, stratified by gram stain negative and positive bacteria status. For statistical association between categorical variables, Chi-square or Fischer’s exact test were used with statistical significance based on p-value ≤ 0.05. Stata (version 14) was used to analyze the data.

### Ethical considerations

The study protocol was approved by the University of Nairobi-Kenyatta National Hospital Ethics Review Committee (Kenya) REF: P687/09/2018; Mulago National Referral Hospital Research and Ethics Committee (Uganda) REF: MREC 1518; and the Uganda National Council of Science and Technology (Uganda) REF: SS4911. Written permission was obtained from hospitals before the commencement of the study. Informed assent was obtained from participants as well as caretakers of minors prior to the study. For minors, assent was obtained after giving them an explanation of the purpose of the study, procedure, and their rights. All data were anonymized and handled confidentially.

## Results

A total of 376 participants with DBWs were enrolled in this study. Table 1 shows a summary of the socio-demographic characteristics of study participants disaggregated according to infection status. Just over half (54%, n = 201) were male, and the median (IQR) age was 18 (22.75) years. The majority (54%) of participants were aged 15 years or older. Dog ownership among bite-patients with DBW was only 11%, and only 5.1% of dogs owned had ever been vaccinated against rabies. Nearly three-quarters (72%) had ever received some information about dogs and dog bite prevention and management. Over half (52.9%, n = 199) of the patients presented with DBWs which were classified as infected.

**Table 1:** Characteristics of the 376 dog bite study participants stratified by the infection status of the wound at initial presentation

		Dog bite wound		
Characteristics	Frequency	Non-infected N = 177 (47.1%)	Infected N = 199 (52.9%)	*p*-value
*Sex*				
Male	201 (53.5)	94 (53.1)	107 (53.8)	
Female	175 (46.5)	83 (46.9)	92 (46.2)	0.898
*Age*				
≤ 15 years	173 (46.0)	82 (46.3)	91 (45.7)	
˃15 years	203 (54.0)	95 (53.7)	108 (54.3)	0.907
*Hospital*				
Entebbe (Wakiso)	110 (29.3)	49 (27.7)	61 (30.7)	
Mulago (Kampala)	266 (70.7)	128 (72.3)	138 (69.3)	0.528
*Religion*				
Christian	301 (80.1)	143 (80.8)	158 (79.4)	
Non-Christian	75 (19.9)	34 (19.2)	41 (20.6)	0.736
*Marital status*				
Not in union	285 (75.8)	144 (81.4)	141 (70.9)	
In union	91 (24.2)	33 (18.6)	58 (29.1)	0.018*
*Highest education level*				
No formal education	53 (14.7)	30 (17.1)	25 (12.6)	
Primary	180 (48.0)	84 (47.7)	96 (48.2)	
Secondary and above	143 (37.3)	62 (35.2)	78 (39.2)	0.432
*Household size*				
≤ 4	176 (46.7)	80 (47.6)	96 (49.7)	
5–8	161 (44.6)	81 (48.2)	80 (41.5)	
≤ 9	24 (6.7)	7 (4.2)	17 (8.8)	0.141
*Employment status*				
No	181 (48.1)	88 (49.7)	93 (47.7)	
Yes	195 (51.9)	89 (50.3)	106 (53.3)	0.563
*Current dog ownership*				
No	334 (88.8)	157 (88.7)	177 (88.9)	
Yes	42 (11.2)	20 (11.3)	22 (11.1)	0.216
*Immunized against rabies*				
No	357 (94.9)	167 (94.3)	190 (95.5)	
Yes	19 (5.1)	10 (5.7)	9 (4.5)	0.618
*Get dog information*				
No	114 (30.3)	57 (32.2)	57 (28.6)	
Yes	262 (69.7)	120 (67.8)	142 (71.4)	0.453
*Socio-economic status*				
Lower	197 (52.5)	92 (52.3)	105 (52.8)	
Middle	62 (16.5)	30 (17.1)	32 (16.0)	
Upper	116 (31.0)	54 (30.7)	62 (31.2)	0.969

The characteristics of the 376 dog bite study participants stratified by infection status of wound at initial presentation.The majority (54%) of participants were aged 15 years or older. Dog ownership among bite-patients with DBW was only 11%, and only 5.1% of dogs owned had ever been vaccinated against rabies. Nearly three-quarters (72%) had ever received some information about dogs and dog bite prevention and management. Over half (52.9%, n = 199) of the patients presented with DBWs which were classified as infected

*Significance at *p* ≤ 0.05

### Characteristics of infected dog bite wounds

Nearly two-thirds (65.3%, 130/199) of the DBWs were single bites, while a third (33.8%) had multiple bites; 15.7% (n = 31) two, and 18.1% (n = 36) more than two bites. Three-quarters (151, 75.9%) of the infected wounds were category II while the rest were category III. The most commonly affected body parts with DBWs were legs (44.4%), followed by thighs (22.2%), head (14.1%), arms (7.1%), and face (2.0%). Notably, legs were the most bitten part, especially among adults of age ˃15 years as shown in Table 2.

**Table 2:** Age-specific distribution of dog bites by body part among the 199 study participants with wound infection

Age (yrs)	Lower limb	Upper limb	Abdomen	Head/face	Combination	Total
≤ 15 years	51	10	8	15	7	91
Percentage	56.1	10.9	8.8	16.5	7.7	100.0
˃15 years	81	4	3	17	3	108
Percentage	75.1	3.7	2.8	15.7	2.8	100.0
Total	132	14	11	32	10	199
Percentage	66.3	7.1	5.5	29.6	5.0	100.0

The Age-specific distribution of dog bites by body part among the 199 participants. Nearly two-thirds (65.3%, 130/199) of the DBWs were single bites, while a third (33.8%) had multiple bites; 15.7% (n = 31) two, and 18.1% (n = 36) more than two bites. Three-quarters (151, 75.9%) of the infected wounds were category II while the rest were category III. The most commonly affected body parts with DBWs were legs (44.4%), followed by thighs (22.2%), head (14.1%), arms (7.1%), and face (2.0%)

### Pre-hospital wound management practices by study participants with infected and non-infected DBWs

Of the 376 study participants, 149 (39.6%) delayed to report to the PET center. However, the differences in the delays between the study participants with infected wounds and those with non-infected wounds were not statistically significant (*p* = 0.277). In addition, only 19.1% (n = 38) of the 199 participants with infected wounds had complied with the pre-clinical guidelines, which included reported washing of the wounds with water and soap and presenting to a healthcare facility within 24 h. Notably, compliance to UCG did not differ between patients with infected wounds and those with non-infected wounds (*p* = 0.800) while the infection rates between those who applied an anticeptic and those who did not, differed significantly (*p* = 0.003). Further, about a quarter of patients who adhered to pre-clinical guidelines (23.7%, 9/38) had applied an antiseptic.

Practices undertaken for patients who did not fully adhere to the pre-clinical guidelines included applying a wide range of materials to the wounds such as herbs, black stone, creams that patients did not know, beans, urine from the biting dog, dust, tobacco, coins, brake fluid, acid, powder made out of dog hair, and salt. Outstandingly, there were two deaths as a result of suspected clinical rabies and both had delayed to present to the healthcare facilities. The details of the health-seeking behaviour of the study participants is described in our previous paper [26]. Table 3 shows a comparison of key pre-hospital wound management practices for patients with non-infected and infected wounds.

**Table 3:** Key pre-hospital wound management practices for the 376 patients with non-infected and infected dog bite wounds

		Dog bite wound		
Practices	Frequency	Non-infected N = 177 (47.1%)	Infected N = 199 (52.9%)	p-value
*Delayed for more than 24 h*				
No	227 (60.4)	112 (63.3)	115 (57.8)	
Yes	149 (39.6)	65 (36.7)	84 (42.2)	0.277
*Washed with water and soap*				
No	204 (55.4)	91 (52.3)	113 (58.3)	
Yes	172 (44.6)	86 (47.7)	86 (41.8)	0.296
Antibiotics administered**				
No	250 (66.5)	122 (68.9)	128 (64.3)	
Yes	126 (33.5)	55 (31.1)	71 (35.7)	0.345
*Antiseptic applied*				
No	330 (87.8)	146 (82.5)	184 (92.5)	
Yes	46 (12.2)	31 (17.5)	15 (7.5)	0.003*
Complied with UCG***				
No	306 (81.4)	145 (81.9)	161 (80.9)	
Yes	70 (18.6)	32 (18.1)	38 (19.1)	0.800

*Significance at p≤0.05

**Antimicrobials administered prior to the patient presenting at the PET center

***The patient had washed the dog bite wound with water and soap in addition to seeking medical care within 24 h

The key pre-hospital wound management practices for the 199 patients with non-infected and infected dog bite wounds. Of the 376 study participants, 149 (39.6%) delayed to report to the PET center. However, the differences in the delays between the study participants with infected wounds and those with non-infected wounds were not statistically significant (*p* = 0.277). In addition, only 19.1% (n = 38) of the 199 participants with infected wounds had complied with the pre-clinical guidelines, which included reported washing of the wounds with water and soap and presenting to a healthcare facility within 24 h. Notably, compliance to UCG did not differ between patients with infected wounds and those with non-infected wounds (*p* = 0.800) while the infection rates between those who applied an anticeptic and those who did not, differed significantly (*p* = 0.003)

### Bacterial isolates from DBWs

**Fig. 1:**
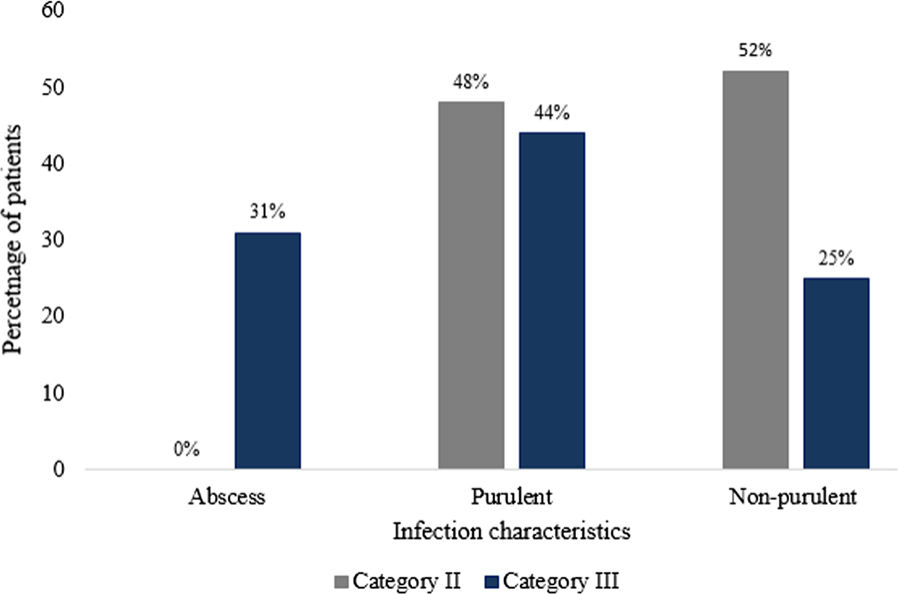
Type of wound infection of category II and III dog bite wounds among the 199 patients

Of the 199 patients with infected DBWs, 151 (75.9%) were in category II, while the rest were in category III. The most common in the category II injuries were the non-purulent wounds (78/151, 52%), while in category III, purulent wounds were the most prevalent (21/48, 44%). The distribution of abscesses, non-purulent and purulent wounds by wound severity is shown in Fig. 1.

Of the 199 DBWs sampled for this study, 168 (84.4%) wounds were culture positive, with 28/151 (18.5%) and 3/48 (6.3%) of the category II and category III respectively, not showing any bacterial growth. A total of 768 isolates were obtained, with gram positive bacteria forming 406 (52.9%) of the yield. Four hundred and ninety six (64.6%) isolates were recovered from category II wounds, while the rest were from category III wounds. Of the 168 swab cultures that showed growth, a total of 123 (73.2%) yielded single cultures, while the rest had a mixture of aerobic and anaerobic bacteria.

Among the 406 g positive bacteria, there were 339 (83.5%) aerobes, of which *Staphylococcus aureus* (103, 30.4%), *Corynebacterium spp* (33, 9.7%), Coagulase-negative staphylococci / CoNS (68, 20.1%), *S. epidermidis* (42, 12.4%), *S. intermedius* (30, 8.8%), and *S. pyogenes* (29, 8.6) were the commonest isolates. Of the 67 anaerobic isolates, *Lactobacillus spp* (31, 46.3%) and *Gemella morbillorium* (21, 31.3%) were the commonest. Furthermore, among the 362 Gram negative isolates, 217 (59.9%) were aerobes and the commonest isolates were *P. maltocida* (64, 29.5%), *Capnocytophaga canimorsus* (36, 16.6%) and *P. canis* (26, 12.0%). However, among the 145 anaerobes, *Fusobacterium spp* (48, 33.1%), *Bacteriodes spp* (34, 23.5%) and *Prevotella spp* (35, 24.1%) were the most frequently isolated bacteria, as shown in Table 4.

**Table 4:** Frequency of bacterial isolates from category II (123 patients) and category III (45 patients) dog bite wounds of patients on initial presentation at 2 PET centers in Uganda

Gram positive bacteria	Number	Percent (%)
*Aerobic gram positive bacteria*		
*Staphylococcus aureus*	103	30.4
*Staphylococcus intermedius*	30	8.8
Coagulase negative Staphylococci	68	20.1
*Streptococuss. canis*	18	5.3
*Streptococuss pyogenes*	29	8.6
Other Streptococci	12	3.5
*Bacillus spp*	11	3.2
*Enterococcus. feacalis*	19	5.6
*Enterococcus. faecium*	2	0.6
Other Enterococci	6	1.8
*Micrococcus spp*	8	2.4
*Corynebacterium spp*	33	9.7
Total: Aerobic gram positive isolates	339	100
*Anaerobic gram positive bacteria*		
Gemella morbillorum*	21	31.3
Lactobacillus spp*	31	46.3
Lactococcus spp*	15	22.4
Total: anaerobic gram positive isolates	67	100
Gram negative bacteria		
*Aerobic Gram negative bacteria*		
*Pasteurella maltocida*	64	29.5
*Pasteurella canis*	26	12.0
Other pasteurella	31	14.3
*Proteus vulgaris*	2	0.9
*Proteus mirabilis*	7	3.2
*Pseudomonas aeuroginosa*	3	1.4
*Pseudomonas stutzeri*	3	1.4
*Pseudomonas alcaligenes*	2	0.9
Other pseudomonas	11	5.1
*Klebsiella pneumonae*	11	5.1
*Klebsiella oxytoca*	6	2.8
*Acinetobacter spp*	3	1.4
*Moellerella wisconsensis*	5	2.3
*Capnocytophaga canimorsus*	36	16.6
*Stenotrophomonas maltophilia*	4	1.8
*Bergeyella zoohelcum*	3	1.4
Total: aerobic gram negative isolates	217	100
*Anaerobic gram negative bacteria*		
Citrobacter werkmanii*	1	0.7
Citrobacter freundii*	2	1.4
E. coli*	6	4.1
Enterobacter asburiae*	1	0.7
Other enterobacter spp*	13	9.0
Serratia rubidae*	2	1.4
Serratia entomophila*	3	2.1
*Fusobacterium spp*	48	33.1
*Bacteriodes spp*	34	23.4
*Prevotella spp*	35	24.1
Total: anaerobic gram negative isolates	145	100

*Facultative anaerobes

Among the 406 g positive bacteria, there were 339 (83.5%) aerobes of which *Staphylococcus aureus* (103, 30.4%), *Corynebacterium spp* (33, 9.7%), Coagulase-negative staphylococci / CoNS (68, 20.1%), *S. epidermidis* (42, 12.4%), *S. intermedius* (30, 8.8%), and *S. pyogenes* (29, 8.6) were the commonest isolates. Of the 67 anaerobic isolates, *Lactobacillus spp* (31, 46.3%) and *Gemella morbillorium* (21, 31.3%) were the commonest. Furthermore, among the 362 Gram negative isolates, 217 (59.9%) were aerobes and the commonest isolates were *P. maltocida* (64, 29.5%), *Capnocytophaga canimorsus* (36, 16.6%) and *P. canis* (26, 12.0%). However, among the 145 anaerobes, *Fusobacterium spp* (48, 33.1%), *Bacteriodes spp* (34, 23.5%) and *Prevotella spp* (35, 24.1%) were the most frequently isolated bacteria

### Antimicrobial susceptibility patterns of bacterial isolates

Table 5 presents the antimicrobial susceptibility patterns of the Gram-positive bacterial isolates. Among the gram positive isolates, the most frequent, *S. aureus*, exhibited high resistance to metronidazole (103, 100%) and oxacillin (94, 91.3%), while the resistance to amoxicillin/clavulanic acid, doxycycline, and trimethoprim / sulfamethoxazole was considerably lower at 19 (18.5%), 14 (13.6%), and 9 (8.7%) respectively. Notably, *S. aureus* was found to be totally sensitive to ceftriaxone, gentamicin, ciprofloxacin, imipenem, streptomycin, doxycycline, methicillin, and chloramphenicol. Among the Streptococci, *S. pyogenes* was the predominant and it was majorly resistant to metronidazole (21, 72.4%) and ceftriaxone (12, 41.4%). Its resistance to imipenem (3, 10.3%) and oxacillin (8, 27.6%) was low, whereas it was sensitive to all other antibiotics. In addition, the most frequent Enterococcus was *E. feacalis* and it exhibited high resistance to methicillin (12, 63.2%), ceftriaxone (11, 58.9%) and metronidazole (19, 100%) while resistance to gentamycin (5, 26.3%) imipenem (4, 21.1%), oxacillin (9, 47.4%), streptomycin (3, 15.8%), and chloramphenicol (7, 36.8%). All *E. feacalis* isolates were susceptible to trimethoprim / sulfamethoxazole, vancomycin, amoxicillin/clavulanic acid, doxycycline, and ciprofloxacin. Notably, all gram positive isolates exhibited total sensitivity to vancomycin and ciprofloxacin.

**Table 5:** Antimicrobial susceptibility patterns of Gram-positive bacterial isolates from wound swab cultures among dog bite patients reporting to two DBW care centers in Uganda in the period April 2019–October 2019

Bacterial isolates	Number of isolates that are resistant to antimicrobial agent, n (%)												
	CRO	ME	CN	AML	SXT	VA	CIP	IPM	S	DOX	OX	C	MET
*Staphylococcus*													
*S. aureus* (n = 103)	0 (0.0)	103 (100)	0 (0.0)	19 (18.5)	9 (8.7)	0 (0.0)	0 (0.0)	0 (0.0)	0 (0.0)	14 (13.6)	94 (91.3)	0 (0.0)	0 (0.0)
*S. intermedius* (n = 30)	0 (0.0)	30 (100)	0 (0.0)	12 (40.0)	0 (0.0)	0 (0.0)	0 (0.0)	0 (0.0)	0 (0.0)	0 (0.0)	28 (93.3)	15 (50.0)	0 (0.0)
CONS (n = 68)	7 (10.3)	66 (97.1)	0 (0.0)	21 (30.9)	0 (0.0)	0 (0.0)	0 (0.0)	0 (0.0)	0 (0.0)	8 (11.8)	61 (89.7)	0 (0.0)	0 (0.0)
*Streptococuss*													
*S. canis* (n = 18)	6 (33.3)	18 (100)	2 (11.1)	0 (0.0)	0 (0.0)	0 (0.0)	0 (0.0)	0 (0.0)	8 (44.4)	18 (100)	4 (22.2)	0 (0.0)	0 (0.0)
*S. pyogenes* (n = 29)	12 (41.4)	21 (72.4)	0 (0.0)	0 (0.0)	0 (0.0)	0 (0.0)	0 (0.0)	3 (10.3)	0 (0.0)	0 (0.0)	8 (27.6)	0 (0.0)	0 (0.0)
Other Streptococci (n = 12)	3 (25.0)	8 (66.7)	0 (0.0)	0 (0.0)	0 (0.0)	0 (0.0)	0 (0.0)	0 (0.0)	0 (0.0)	0 (0.0)	2 (16.7)	0 (0.0)	0 (0.0)
*Bacillus spp* (n = 11)	4 (36.4)	11 (100)	3 (27.3)	1 ((9.1)	4 (36.4)	0 (0.0)	0 (0.0)	0 (0.0)	0 (0.0)	0 (0.0)	3 (27.3)	2 (18.1)	0 (0.0)
*Enterococcus*													
*E. feacalis* (n = 19)	11 (58.9)	19 (100)	5 (26.3)	0 (0.0)	0 (0.0)	0 (0.0)	0 (0.0)	4 (21.1)	3 (15.8)	0 (0.0)	9 (47.4)	7 (36.8)	12 (63.2)
*E. faecium* (n = 2)	1 (50.0)	2 (100)	0 (0.0)	0 (0.0)	0 (0.0)	0 (0.0)	0 (0.0)	0 (0.0)	0 (0.0)	1 (50.0)	1 (50.0)	0 (0.0)	0 (0.0)
Other Enterococci (n = 6)	0 (0.0)	6 (100)	1 (16.7)	0 (0.0)	0 (0.0)	0 (0.0)	0 (0.0)	0 (0.0)	1 (16.7)	0 (0.0)	2 (33.3)	0 (0.0)	0 (0.0)
*Micrococcus spp* (n = 8)	0 (0.0)	8 (100)	0 (0.0)	0 (0.0)	0 (0.0)	0 (0.0)	0 (0.0)	0 (0.0)	0 (0.0)	0 (0.0)	0 (0.0)	0 (0.0)	0 (0.0)
*Corynebacterium spp* (n = 33)	0 (0.0)	33 (100)	9 (27.3)	0 (0.0)	0 (0.0)	0 (0.0)	0 (0.0)	0 (0.0)	0 (0.0)	0 (0.0)	12 (36.4)	0 (0.0)	0 (0.0)
*Gemella morbillorum* (n = 21)	0 (0.0)	21 (100)	1 (4.8)	0 (0.0)	0 (0.0)	0 (0.0)	0 (0.0)	0 (0.0)	2 (9.5)	0 (0.0)	18 (85.7)	0 (0.0)	0 (0.0)
*Lactobacillus spp* (n = 31)	8 (25.8)	26 (83.9)	5 (16.1)	4 (12.9)	0 (0.0)	0 (0.0)	0 (0.0)	8 (25.8)	7 (22.6)	6 (19.4)	26 (100)	8 (25.8)	0 (0.0)
*Lactococcus spp* (n = 15)	8 (53.3)	8 (53.3)	3 (20.0)	0 (0.0)	3 (20.0)	0 (0.0)	0 (0.0)	0 (0.0)	0 (0.0)	4 (26.7)	10 (66.7)	4 (26.7)	2 (13.3)
Total isolates N = 406	60 (14.8)	380 (93.6)	29 (7.1)	57 (14.0)	16 (3.9)	0 (0.0)	0 (0.0)	15 (3.7)	21 (5.2)	51 (12.6)	278 (68.5)	36 (8.9)	14 (3.5)

*CRO* ceftriaxone; *ME* metronidazole; *CN* gentamycin; *AML* amoxicillin / clavulanic acid; *SXT* trimethoprim / sulfamethoxazole; *VA* vancomycin; *CIP* ciprofloxacin; *IPM* imipenem; *S* streptomycin; *DOX* doxycycline; *OX* oxacillin; *C* chloramphenicol; *MET* methicillin

The antimicrobial susceptibility patterns of the Gram-positive bacterial isolates. Among the gram positive isolates, the most frequent, *S. aureus*, exhibited high resistance to metronidazole (103, 100%) and oxacillin (94, 91.3%) while the resistance to amoxicillin, doxycycline and trimethoprim / sulfamethoxazole was considerably lower at 19 (18.5%), 14 (13.6%) and 9 (8.7%), respectively

In Table 6, the antimicrobial susceptibility patterns of the Gram-negative bacterial isolates from DBWs are shown. The predominant gram negative isolates were *P. maltocida* (n = 64), *P. canis* (n = 26) and *Capnocytophaga canimorsus* (n = 36). *P. maltocida* was highly resistant to metronidazole (64, 100%) but had low resistance to gentamycin (6, 9.4%), amoxicillin/clavulanic acid (12, 18.8%), ampicillin (8, 12.5%) and oxacillin (6, 9.4%). It was susceptible to all other antimicrobial drugs. However, much as *P. canis* was highly resistant to metronidazole (26, 100%), the resistance to amoxicillin/clavulanic acid (7, 26.9%), and ampicillin (10, 38.5%) was substantially low. Nevertheless, the *P. canis* isolates were sensitive to the rest of the antimicrobials, including ceftriaxone, gentamicin, trimethoprim / sulfamethoxazole, ciprofloxacin, imipenem, doxycycline, and oxacillin. *Capnocytophaga canimorsus* isolates were resistant to metronidazole (36, 100%), oxacillin (34, 94.4%), ampicillin (31, 86.1%), amoxicillin/clavulanic acid (16, 44.4%), trimethoprim / sulfamethoxazole (15, 41.7%), ceftriaxone (11, 30.6%), chloramphenicol (10, 27.8%), and streptomycin (5, 13.9%). *Prevotella spp* isolates were also 100% resistant to metronidazole but its resistance to amoxicillin/clavulanic acid and doxycycline was noticeably low i.e., 6 (17.1%) and 12 (34.3%), respectively. Notably, all E. coli isolates were resistant to metronidazole, amoxicillin/clavulanic acid, doxycycline, trimethoprim / sulfamethoxazole, oxacillin, and ampicillin. Conspicuously, all isolates were resistant to metronidazole but susceptible to ciprofloxacin while one isolate (*P. alcaligenes)* was resistant to imipenem.

**Table 6:** Antimicrobial susceptibility patterns of Gram-negative bacterial isolates from wound swab cultures among dog bite patients reporting to two DBW care centers in Uganda in the period March 2019–October 2019

Bacteria	Number of isolates that are resistant to antimicrobial agent, n (%)											
	CRO	MET	CN	AML	SXT	CIP	IPM	S	DOX	OX	C	AMP
*Pasteurella*												
*P. maltocida* (n = 64)	0 (0.0)	64 (100.0)	0 (0.0)	12 (18.8)	0 (0.0)	0 (0.0)	0 (0.0)	0 (0.0)	0 (0.0)	6 (9.4)	0 (0.0)	8 (12.5)
*P. canis* (n = 26)	0 (0.0)	26 (100.0)	0 (0.0)	7 (26.9)	0 (0.0)	0 (0.0)	0 (0.0)	0 (0.0)	0 (0.0)	0 (0.0)	0 (0.0)	10 (38.5)
Other pasteurella (n = 31)	0 (0.0)	31 (100.0)	3 (9.7)	0 (0.0)	0 (0.0)	0 (0.0)	0 (0.0)	0 (0.0)	0 (0.0)	0 (0.0)	0 (0.0)	0 (0.0)
*Proteus*												
*P. vulgaris* (n = 2)	0 (0.0)	2 (100.0)	0 (0.0)	2 (100.0)	0 (0.0)	0 (0.0)	0 (0.0)	0 (0.0)	2 (100.0)	0 (0.0)	0 (0.0)	0 (0.0)
*P. mirabilis* (n = 7)	0 (0.0)	7 (100.0)	0 (0.0)	0 (0.0)	0 (0.0)	0 (0.0)	0 (0.0)	3 (42.9)	4 (57.1)	2 (28.6)	0 (0.0)	3 (42.9)
*Pseudomonas*												
*P. aeuroginosa* (n = 3)	0 (0.0)	3 (100.0)	0 (0.0)	0 (0.0)	0 (0.0)	0 (0.0)	0 (0.0)	0 (0.0)	0 (0.0)	0 (0.0)	0 (0.0)	3 (100.0)
*P. stutzeri* (n = 3)	0 (0.0)	3 (100.0)	0 (0.0)	1 (33.3)	0 (0.0)	0 (0.0)	0 (0.0)	0 (0.0)	0 (0.0)	3 (100.0)	0 (0.0)	0 (0.0)
*P. alcaligenes* (n = 2)	1 (50.0)	2 (100.0)	1 (50.0)	1 (50.0)	0 (0.0)	0 (0.0)	1(50.0)	2 (100.0)	2 (100.0)	2 (100.0)	0 (0.0)	2 (100.0)
Other pseudomonas* (n = 11)	0 (0.0)	11 (100.0)	0 (0.0)	0 (0.0)	0 (0.0)	0 (0.0)	0 (0.0)	3 (27.3)	0 (0.0)	11 (100.0)	2 (18.1)	3 (27.3)
*Klebsiella*												
*K. pneumonae* (n = 11)*	2 (18.1)	11 (100.0)	0 (0.0)	11 (100.0)	5 (45.5)	0 (0.0)	0 (0.0)	0 (0.0)	7 (63.6)	11 (100.0)	3 (27.3)	11 (100.0)
*K. oxytoca* (n = 6)	0 (0.0)	6 (100.0)	4 (66.7)	6 (100.0)	4 (66.7)	0 (0.0)	0 (0.0)	0 (0.0)	0 (0.0)	6 (100.0)	0 (0.0)	6 (100.0)
*Acinetobacter spp* (n = 3)	0 (0.0)	3 (100.0)	0 (0.0)	0 (0.0)	3(100.0)	0 (0.0)	0 (0.0)	0 (0.0)	0 (0.0)	3 (100.0)	0 (0.0)	3 (100.0)
*Moellerella wisconsensis* (n = 5)	3 (60.0)	5 (100.0)	0 (0.0)	5 (100.0)	3 (60.0)	0 (0.0)	0 (0.0)	0 (0.0)	3 (60.0)	5 (100.0)	2 (40.0)	5 (100.0)
*Capnocytophaga canimorsus* (n = 36)	11 (30.6)	36 (100.0)	0 (0.0)	16 (44.4)	15 (41.7)	0 (0.0)	0 (0.0)	5 (13.9)	0 (0.0)	34 (94.4)	10 (27.8)	31 (86.1)
*Stenotrophomonas maltophilia* (n = 4)	0 (0.0)	4 (100.0)	1 (25.0)	0 (0.0)	0 (0.0)	0 (0.0)	0 (0.0)	0 (0.0)	0 (0.0)	0 (0.0)	0 (0.0)	0 (0.0)
*Bergeyella zoohelcum* (n = 3)	0 (0.0)	3 (100.0)	0 (0.0)	3 (100.0)	3 (100.0)	0 (0.0)	0 (0.0)	3 (100.0)	3 (100.0)	0 (0.0)	0 (0.0)	3 (100.0)
*Citrobacter*												
*C. werkmanii* (n = 1)	0 (0.0)	1 (100.0)	0 (0.0)	0 (0.0)	0 (0.0)	0 (0.0)	0 (0.0)	0 (0.0)	1 (100.0)	0 (0.0)	0 (0.0)	1 (100.0)
*C. freundii* (n = 2)	2 (100.0)	2 (100.0)	0 (0.0)	1 (50.0)	2 (100.0)	0 (0.0)	0 (0.0)	0 (0.0)	1 (100.0)	1 (100.0)	2 (100.0)	2 (100.0)
*E. coli* (n = 6)	0 (0.0)	6 (100.0)	0 (0.0)	4 (66.7)	6 (100.0)	0 (0.0)	0 (0.0)	0 (0.0)	4 (66.7)	6 (100.0)	0 (0.0)	4 (66.7)
*Enterobacter*												
*E. asburiae* (n = 1)	0 (0.0)	1 (100.0)	0 (0.0)	0 (0.0)	0 (0.0)	0 (0.0)	0 (0.0)	0 (0.0)	1 (100.0)	1 (100.0)	1 (100.0)	0 (0.0)
Other enterobacter spp (n = 13)	7 (53.9)	13 (100.0)	0 (0.0)	6 (46.2)	13 (100.0)	0 (0.0)	0 (0.0)	0 (0.0)	7 (53.9)	13 (100.0)	0 (0.0)	13 (100.0)
*Serratia*												
*S. rubidae* (n = 2)	0 (0.0)	2 (100.0)	0 (0.0)	2 (100.0)	2 (100.0)	0 (0.0)	0 (0.0)	0 (0.0)	0 (0.0)	0 (0.0)	0 (0.0)	0 (0.0)
*S. entomophila* (n = 3)	0 (0.0)	3 (100.0)	0 (0.0)	0 (0.0)	0 (0.0)	0 (0.0)	0 (0.0)	0 (0.0)	0 (0.0)	0 (0.0)	0 (0.0)	3 (100.0)
*Fusobacterium spp* (n = 48)	0 (0.0)	48 (100.0)	0 (0.0)	0 (0.0)	0 (0.0)	0 (0.0)	0 (0.0)	0 (0.0)	8 (16.7)	0 (0.0)	0 (0.0)	0 (0.0)
*Bacteriodes spp* (n = 34)	0 (0.0)	34 (100.0)	4 (11.8)	1 (2.9)	0 (0.0)	0 (0.0)	0 (0.0)	0 (0.0)	28 (82.4)	2 (5.9)	0 (0.0)	0 (0.0)
*Prevotella spp* (n = 35)	0 (0.0)	35 (100.0)	0 (0.0)	6 (17.1)	0 (0.0)	0 (0.0)	0 (0.0)	0 (0.0)	12 (34.3)	0 (0.0)	0 (0.0)	0 (0.0)
Total isolates N = 362	26 (7.2)	362 (100)	13 (5.3)	54 (14.9)	53 (14.6)	0 (0.0)	1 (0.3)	16 (4.4)	83 (22.9)	106 (29.3)	20 (5.5)	111 (30.7)

*CRO* ceftriaxone; *ME* metronidazole; *CN* gentamycin; *AML* amoxicillin / clavulanic acid; *SXT* trimethoprim / sulfamethoxazoleI; *VA* vancomycin; *CIP* ciprofloxacin; *IPM* imipenem; *S* streptomycin; *DOX* doxycycline; *OX* oxacillin; *C* chloramphenicol; *AMP* ampicillin

The antimicrobial susceptibility patterns of the Gram-negative bacterial isolates from DBWs. The predominant gram negative isolates were *P. maltocida* (n = 64), *P. canis* (n = 26) and *Capnocytophaga canimorsus* (n = 36). *P. maltocida* was highly resistant to metronidazole (64, 100%) but low resistance to gentamycin (6, 9.4%), amoxicillin (12, 18.8%), ampicillin (8, 12.5%) and oxacillin (6, 9.4%). It was susceptible to all other antimicrobial drugs. However, much as *P. canis* was highly resistant to metronidazole (26, 100%), the resistance to amoxicillin (7, 26.9%), and ampicillin (10, 38.5%) was substantially low

Generally, there was not much difference between the resistance of bacterial isolates obtained from category II and category II DBWs. However, for gram-positive isolates from category III wounds, there was more resistance to many drugs compared to category II wounds. As shown in Table 7, resistance to streptomycin (*p* = 0.001), doxycycline (*p* = 0.038), and oxacillin (*p* = 0.0001) was significantly associated with the isolate being from category III DBWs among gram positive bacteria. Furthermore, for gram negative bacteria, there was no significant association between the resistance of isolates from categories II and III DBWs.

**Table 7:** Comparison of antimicrobial resistant patterns of Gram-positive and Gram-negative isolates among patients with category II and category III DBW reporting to 2 PET centers in Uganda between April and October 2019

Antimicrobial	Pattern	Gram positive isolates			Gram negative isolates		
		Category II wounds (n = 279), %	Category III wounds (n = 127), %	*X*^*2*^ (p-value)	Category II wounds n = 217	Category III wounds n = 145	*X*^*2*^ (p-value)
Ceftriaxone	R	38 (13.6)	22 (17.3)	1.23 (0.54)	15 (6.9)	11 (7.6)	1.85 (0.40)
	I	7 (2.5)	2 (1.6)		3 (1.4)	5 (3.5)	
	S	234 (83.9)	103 (81.1)		199 (91.7)	128 (88.9)	
Metronidazole*	R	264 (94.6)	116 (91.3)	1.57 (0.21)	217 (100.0)	145 (100.0)	–
	I	15 (5.4)	11 (8.7)		0 (0.0)	0 (0.0)	
	S	0 (0.0)	0 (0.0)		0 (0.0)	0 (0.0)	
Gentamicin	R	21 (7.6)	8 (6.3)	1.97 (0.37)	9 (4.2)	4 (2.8)	4.24 (0.11)
	I	11 (3.9)	9 (7.1)		3 (1.4)	7 (4.8)	
	S	247 (88.5)	110 (86.6)		205 (94.5)	134 (92.4)	
Amoxicillin	R	34 (12.2)	23 (18.1)	2.54 (0.11)	31 (14.3)	23 (15.9)	0.16 (0.69)
	S	245 (87.8)	104 (81.9)		185 (85.7)	122 (84.1)	
Trimethoprim / sulfamethoxazole	R	11 (3.9)	5 (3.9)	0.14 (0.93)	37 (17.0)	16 (11.0)	4.74 (0.09)
	I	7 (2.5)	4 (3.2)		9 (4.2)	12 (8.3)	
	S	261 (93.6)	118 (92.9)		171 (78.8)	117 (80.7)	
Vancomycin*	R	0 (0.0)	0 (0.0)	–	ND	ND	–
	S	279 (100.0)	127 (100.0)		ND	ND	
Ciprofloxacin	R	0 (0.0)	0 (0.0)	–	0 (0.0)	0 (0.0)	–
	S	279 (100.0)	127 (100.0)		217 (100.0)	145 (100.0)	
Imipenem*	R	9 (3.3)	6 (4.7)	0.55 (0.46)	1 (0.5)	0 (0.0)	
	S	270 (96.7)	121 (95.3)		216 (99.5)	145 (100.0)	
Streptomycin	R	7 (2.5)	14 (11.0)	13.51 (0.001)**	6 (2.8)	10 (6.9)	3.84 (0.15)
	I	4 (1.4)	3 (2.4)		5 (2.3)	2 (1.4)	
	S	268 (96.1)	110 (86.6)		206 (94.9)	133 (91.7)	
Doxycycline	R	23 (8.3)	14 (11.0)	6.56 (0.038)**	55 (25.3)	28 (19.3)	329 (0.19)
	I	4 (1.4)	7 (5.5)		9 (4.2)	3 (2.1)	
	S	252 (90.3)	106 (83.5)		153 (70.5)	114 (78.6)	
Oxacillin*	R	169 (60.6)	109 (85.8)	25.78 (≤ 0.0001)**	62 (28.6)	44 (30.3)	0.13 (0.72)
	S	110 (39.4)	18 (14.2)		155 (71.4)	101 (69.7)	
Chloramphenicol	R	22 (7.9)	14 (11.1)	3.14 (0.21)	12 (5.5)	8 (5.5)	3.57 (0.17)
	I	31 (11.1)	20 (15.7)		5 (2.3)	9 (6.2)	
	S	226 (81.0)	93 (73.2)		200 (92.2)	128 (88.3)	
Methicillin*	R	9 (3.2)	5 (3.9)	0.27 (0.61)	ND	ND	–
	S	270 (96.8)	122 (96.1)		ND	ND	
Ampicillin	R	ND	ND	–	68 (31.3)	43 (29.7)	1.19 (0.55)
	I	ND	ND		7 (3.2)	8 (5.5)	
	S	ND	ND		142 (65.4	94 (64.8)	

*S* sensitive; *R* resistant; *I* intermediate; *ND* not done

^*^Antimicrobial agent did not have an intermediate zone; **differences are statistically significant at p ≤ 0.05

That resistance to streptomycin (*p* = 0.001), doxycycline (*p* = 0.038), and oxacillin (*p* = 0.0001) was significantly associated with the isolate being from category III DBWs among gram positive isolates. For gram negative isolates, there were no significant association between the resistance of isolates of categories II and III DBWs

### Multidrug resistance of bacterial isolates

Out of the 768 isolates, 226 (29.4%) were resistant to at least one antimicrobial in three or more antimicrobial classes. Thus, they were taken to be multidrug resistant (MDR). As shown in Additional file 2, among the 406 g positive isolates, 121/406 (29.8%) were found to be multidrug resistant. Specifically, these included*, S. intermedius, S. canis,* and *Corynebacterium spp* which were resistant to three classes of antimicrobial agents. In contrast, *S. aureus, S. pyogenes, E. feacalis, Lactobacillus spp* and *Lactococcus spp* were resistant to 4 or more classes of antimicrobial drugs. Further, of the 362 g-negative isolates, 105 (29.0%) exhibited MDR. Of these, *P. vulgaris, C. werkmanii, E. asburiae,* and *Bacteriodes spp* were resistant to antimicrobial agents in three classes. Additionally, *P. mirabilis, K. pneumonae, K. oxytoca, Moellerella wisconsensis, Capnocytophaga canimorsus, E. coli,* and *Bergeyella zoohelcum,* were resistant to were resistant to 4 or more classes of antimicrobial drugs as shown in Table 8.

**Table 8:** Antimicrobial resistance patterns of multidrug resistant bacterial pathogens isolated from wound swab cultures among patients with DBW attending PET centers in Uganda

Bacteria	Antimicrobial classes and related number of resistant isolates (%)				
Gram positive (n = 406)	Number	R_1_	R_2_	R_3_	≥ R_4_
Staphylococcus aureus*	103 (25.4)	56 (13.8)	11 (2.7)	24 (5.8)	12 (2.9)
Staphylococcus intermedius*	30 (7.4)	2 (6.7)	15 (3.7)	13 (3.2)	0 (0.0)
Streptococuss canis*	18 (4.4)	0 (0.0)	2 (0.5)	10 (2.5)	6 (1.5)
Streptococuss pyogenes*	29 (7.1)	2 (0.5)	14 (3.5)	12 (2.9)	1 (0.3)
Bacillus spp*	11 (2.7)	2 (0.5)	3 (0.7)	4 (1.0)	2 (0.5)
Enterococcus feacalis*	19 (4.7)	4 (1.0)	9 (2.2)	2 (0.5)	4 (1.0)
*Enterococcus faecium*	2 (0.5)	0 (0.0)	1 (0.3)	1 (0.3)	0 (0.0)
*Corynebacterium spp*	33 (8.1)	18 (4.4)	9 (2.2)	6 (1.5)	0 (0.0)
Lactobacillus spp*	31 (7.6)	9 (2.2)	8 (1.9)	4 (1.0)	10 (2.5)
Lactococcus spp*	23 (5.6)	7 (1.8)	6 (1.5)	6 (1.5)	4 (1.0)
Total MDR isolates				82 (20.2)	39 (9.6)
*Gram negative (n* = *362)*					
*Proteus vulgaris*	2 (0.6)	0 (0.0)	0 (0.0)	2 (0.6)	0 (0.0)
*Proteus mirabilis*	7 (1.9)	2 (0.6)	1 (0.3)	2 (0.6)	2 (0.6)
*Pseudomonas alcaligenes*	2 (0.6)	0 (0.0)	0 (0.0)	0 (0.0)	2 (0.6)
Other pseudomonas	11 (3.0)	4 (1.1)	3 (0.8)	2 (0.6)	2 (0.6)
*Klebsiella pneumonae*	11 (3.0)	0 (0.0)	3 (0.8)	4 (1.1)	4 (1.1)
*Klebsiella oxytoca*	6 (1.7)	0 (0.0)	2 (0.6)	0 (0.0)	4 (1.1)
*Acinetobacter spp*	3 (0.8)	0 (0.0)	0 (0.0)	3 (0.8)	0 (0.0)
*Moellerella wisconsensis*	5 (1.4)	0 (0.0)	1 (0.3)	1 (0.3)	3 (0.8)
*Capnocytophaga canimorsus*	36 (9.9)	3 (0.8)	8 (2.2)	11 (3.0)	14 (3.9)
*Bergeyella zoohelcum*	3 (0.8)	0 (0.0)	0 (0.0)	0 (0.0)	3 (0.8)
*Citrobacter werkmanii*	1 (0.3)	0 (0.0)	0 (0.0)	1 (0.3)	0 (0.0)
*Citrobacter freundii*	2 (0.6)	0 (0.0)	0 (0.0)	0 (0.0)	2 (0.6)
*Enterobacter asburiae*	1 (0.3)	0 (0.0)	0 (0.0)	1 (0.3)	0 (0.0)
Other enterobacter spp	13 (3.6)	0 (0.0)	0 (0.0)	6 (1.7)	7 (1.9)
*Bacteriodes spp*	34 (9.4)	5 (1.4)	4 (1.1)	25 (6.9)	0 (0.0)
Total MDR isolates				58 (16.0)	47 (12.9)

*MDR bacteria; R1–** ≥ **R4 Resistance to classes of antimicrobial agents 1, 2, 3, 4 and above

The antimicrobial resistance patterns of multidrug resistant bacterial pathogens isolated from wound swab cultures among patients with DBW attending PET centers in Uganda. Out of the 768 isolates, 226 (29.4%) were resistant to at least one antimicrobial in three or more antimicrobial classes. Thus, they were taken to be multidrug resistant (MDR)

## Discussion

This study aimed at describing the bacteriology of dog bites and evaluating the sensitivity of the bacterial isolates from such wounds to antimicrobial agents that are commonly used DBW management. It was found that approximately half of the patients presented with infected wounds and nearly 85% of the swabs taken yielded cultures, especially those from category II wounds. Additionally, the most frequently isolated bacteria were *Staphylococcus aureus*, Coagulase-negative staphylococci, *Corynebacterium spp*, *P. maltocida*, *Capnocytophaga canimorsus* and *P. canis*. The isolates were majorly resistant to metronidazole, oxacillin, ampicillin and doxycycline while all gram-positive isolates were susceptible to vancomycin and ciprofloxacin.

In the study, 52.9% of the patients presented with infected DBWs. This is in contrast with the majority of studies which have put the infection rates of DBWs between 5 and 25% [11, 30]. However, it should be noted that the risk of infection depends on the nature and site of the wound as well as on individual patient characteristics. Therefore, the differences in study populations and settings might explain the variance in the infection rates between this and other studies. In addition, the infections being purulent in 54% and non-purulent in 23% of the participants of this study is comparable to different studies elsewhere. Although they used small sample sizes, these studies found that the purulence and non-purulence of DBWs were at 58% and 30%, respectively [10].

Contamination of DBWs results from the oral microflora of dogs as well as the environment. Therefore, a variety of organisms that generally result from the aerobic and anaerobic microbial flora of the oral cavity of the dog and the patient’s own skin flora can be recovered from bite wounds. In this study, 84.4% of the swabs were culture positive, an outcome that is similar to other wound studies in Ethiopia and Nigeria [31, 32], though lower in others in similar settings [33]. Furthermore, in this study, 73% of the wounds yielded monomicrobial growth, while the rest had a mixture of aerobic and anaerobic bacteria. This result is lower than that found in other wound studies, though only slightly [31, 34], but higher than the 48% reported by Talan et al. [10]. The rates of isolation in this study were 72.4% and 22.6% for aerobic and anaerobic bacteria, respectively. Yielding more aerobic isolates is similar to earlier studies on dog bites [21], although other studies have isolated more anaerobic than aerobic bacteria [31].

Staphylococci, streptococci, and corynebacterium were the most common aerobic isolates. The most predominant gram positive aerobe was *S. aureus* at 30.4% of such aerobes. The isolation rate is just slightly higher than that obtained in similar wound studies in Nigeria and Italy [35, 36]. The slight differences of less than 5% may be explained by the different settings where the comparative studies were conducted in hospital settings on surgical wounds. Nonetheless, the rate in this study is lower than that reported in Ethiopia [31, 37]. Together with *S. pyogenes* which was also fairly common, *Staphylococcus aureus* is one of the organisms often considered responsible for cellulitis in wounds. These bacteria are rarely found in the dog oral cavity, and are considered part of normal skin flora [13]. However, the 8.8% rate of *S. intermedius* is higher than in previous isolations which had rates of 2% but lower than other wound studies which yielded the bacteria at 12% of the total isolates [13, 15].

For gram negative bacteria*, Pasteurella spp* were the most dominant. In this study, *P. maltocida* was the most frequently isolated bacteria. This is significantly different from other reports that have identified *P. canis* was the predominant isolate from dog bites [10, 38]. In addition, the prominence of *Pasteurella* contradicts earlier impressions that this it is an uncommon pathogen in dog bites injuries [39, 40]. However, our findings are in agreement with previous studies which identified *P. maltocida* as being predominant over other species of *Pasteurella* [15]. Our findings nonetheless support the findings that *Pasteurella species* are among the most common canine oropharyngeal isolates, isolated in 12.5–87% of canines. Therefore, our data upholds *Pasteurella*’s reputation for pathogenicity and relevance in DBW infection [10]. Importantly, although most species of *Pasteurella* are taken to be normal flora of animal saliva, *P. canis* is distinctive because it is found only in the oral cavities of dogs. Having isolated some of it in wounds of patients that had complied with pre-hospital guidelines brings into question the efficiency of the application of the standard recommendations.

In this study, there were 36 isolates of *Capnocytophaga canimorsus.* This bacterium has been frequently reported as a common cause of serious infection associated with dog bites in humans [41, 42]. It has been described as normal flora in 75% of the oral cavities of dogs [43] and its association with severe infection following DBWs has been well described. It is therefore not surprising that it was possible to isolate it from mainly patients who had not washed their wounds prior to presentation at the PET centers. Furthermore, the most common gram negative anaerobes in this study included *Fusobacterium spp*, *Bacteroides spp*, and *Prevotella spp*. These anaerobes have also been isolated elsewhere and identified as predominant [15, 40]. However, they are not known to be of any zoonotic significance but they are thought to originate from the oral cavity of dogs.

The use of antibiotics in animal bite wounds is surrounded by considerable controversy. Much as some authors have found antimicrobial agents to be useful [16], others have argued that they are not prophylactically effective [17, 44]. It is for this reason that some studies have recommended antimirobial agents for therapeutic and not prophylactic purposes [45]. Much as this is the case, the UCG still call for their prophylactic use in DBW with a high risk of infection. However, for therapy, it is recommended that selection of an appropriate antimicrobial agent should be based on cultures from infected wounds, followed by antimicrobial susceptibility testing. This is why antibiotics, including metronidazole, methicillin, amoxicillin/clavulanic acid, doxycyline and cotrimoxazole are recommended in UCG but with the caveat that they are used after culture and sensitivity tests [22]. Nonetheless, such tests are not routinely performed for patients in clinical practice in the entire country. Missing the benefits of sensitivity tests poses significant risks to patients in terms of finances, side effects and development of antimicrobial resistance [46]. Already, the latter has been widely reported in dog bite wounds [15, 47].

Amoxicillin/clavulanic acid is the first-choice agent both for prophylaxis and treatment for DBW patients who are not penicillin allergic [48]. The present study demonstrated that amoxicillin/clavulanic acid was resistant to only 14% of the isolates, which was lower in some earlier studies [31, 49]. This study is therefore in agreement with other authors who have suggested that amoxicillin/clavulanic acid is one of the most effective antibiotic treatments for a dog bite as it covers the most likely polymicrobial aerobic and anaerobic organisms that infect dog bite wounds [50]. Besides, in older animal bite wounds, presenting 9–24 h after injury, amoxicillin/clavulanic acid has been reported to reduce the infection rate significantly [51]. However, the observed differences in the levels of susceptibility between amoxicillin/clavulanic acid and oxacillin may require further investigation. Furthermore, beyond UCG, Metronidazole is recommended to treat infection in DBWs [18, 44] especially for those allergic to penicillin [48]. In this study, all isolates were resistant to metronidazole which is known to be generally effective against Gram-negative anaerobes. This finding is in conflict with some studies which have found it effective in treating anaerobic infections including skin and soft tissues [52]. However, in Tanzania, metronidazole had a questionable activity in treating wound infection when compared to other studies, especially in bacteria isolated from the head and neck and other parts of the body [53].

In this study, isolation of some MRSA may support the growing concerns sorrounding the role of community-associated methicillin-resistant *S. aureus* (CA-MRSA) in skin and soft-tissue infections as well as whether MRSA is a key pathogen in infections following animal bites [54]. The isolation of MRSA from dog bite wounds is not surprising because several studies have reported its existence in dogs [55, 56]. Perhaps what is more concerning is that MRSA-associated infections in dogs and other pets are typically acquired from their owners and can potentially cycle between such animals and their human acquaintances [14, 57]. Worse still, some of the dogs carrying the bacteria may remain healthy thus the potential for undetected transmission [58].

Just like in this study, gram positive and gram negative bacteria resistant to trimethoprim / sulfamethoxazole have been isolated before from animal bites as well as other wounds [31, 59]. In addition, in this study, 29% of the isolates were multidrug resistance (MDR). This is in contrast with other studies that have found multidrug resistance to be as high as 70–95% [37]. However, some of the MDR isolates like *P. mirabilis* had been reported before to be in circulation in Uganda [60]. The presence of such bacteria in Uganda may be due to the continued massive reliance on antimicrobials as a first-hand treatment option by physicians, hence propagation of more resistant strains of the bacteria.

## Conclusions

The infection rates for DBWs in Uganda are higher than those reported elsewhere.  This indicates a critical need for further studies to identify infection prevention and control measures that can efficiently decrease the rate of DBW infection with a strategic aim of reducing the use of antimicrobial agents. Further, *Staphylococcus aureus,* CONS, *Corynebacterium spp, Gemella morbillorium, Lactobacillus spp, Pasteurella spps,* and *Capnocytophaga canimorsus* are the most frequently involved pathogen in the infection of DBWs. Since some isolates like *P. canis* are known to be exclusively from oral cavities of biting dogs, their isolation in patients that had reported complying with pre-hospital guidelines, brings into question how the recommendations in the guidelines are implemented. Among the antimicrobials recommended in the UCG for the treatment of DBWs, metronidazole showed the highest resistance, even for Gram-negative anaerobes, and there is a high rate of MDR to antibiotics commonly used to treat DBWs. We recommend that UCG offers details how wounds should be washed during first aid, in addition to more studies being undertaken into metronidazole to decide whether it is still useful in treatment of animal bites. Lastly, DBWs should also be included in the continuous surveillance of antimicrobial resistance during the routine AMR programs to encourage rational use of antimicrobial agents.

## Supplementary Information

**Additional file 1.** The questionnaire used to collect data on factors associated with pre-clinical care practices undertaken for dog bite patients. The variables include patient socio-demographic factors, dog factors and circumstacnes sorrounding the bite event before, during and after it happened.

**Additional file 2.** Summary of antimicrobial resistance patterns of bacterial pathogens isolated from wound swab cultures among patients with DBWs attending PET centers in Uganda.

## Data Availability

All data generated or analysed during this study are included in this published article. The datasets are available from the corresponding author on reasonable request.

## Abbreviations

PET: Post-exposure therapy
DBW: Dog bite wound
MDR: Multidrug resistance
AMR: Antimicrobial resistance
UCG: Uganda Clinical Guidelines
CA-MRSA: Community-associated methicillin-resistant *S. aureus*
MRSA: Methicillin-resistant *S. aureus*
