# Supplementary material for: The potential for the double risk of rabies and antimicrobial resistance in a high rabies endemic setting: detection of antibiotic resistance in bacterial isolates from infected dog bite wounds in Uganda
Source: Antimicrob Resist Infect Control. 2022 Nov 13;11:142. doi: 10.1186/s13756-022-01181-0 (PMC9655799; doi:10.1186/s13756-022-01181-0)
Supplement: Supplementary file 1 — Additional file 1. The questionnaire used to collect data on factors associated with pre-clinical care practices undertaken for dog bite patients. The variables include patient socio-demographic factors, dog factors and circumstacnes sorrounding the bite event before, during and after it happened. [file 13756_2022_1181_MOESM1_ESM.docx]

## PART A: QUESTIONNAIRE ON FACTORS ASSOCIATED WITH PRE-CLINICAL CARE PRACTICES UNDERTAKEN BY DOG BITE PATIENTS

**English version**

**Date (DD/MM/YY**) ________________________________________

**Time respondent had reported to hospital:** ___________________________________

**A. Research site / Hospital name:** _________________________Hospital code: ___________

**B. Respondent details**

Name code of patient: _______________________

Residence codes: Village___________________Sub county ___________________________

Landmark to home (please describe): _____________________________________________

Contact details: Telephone 1___________________________ Telephone 2________________

Name of next of kin: Surname _____________________ last name _______________________

Contact details of next of kin: Telephone 1_________________ Telephone 2________________

Date of birth: Can tell ❑ Cannot remember ❑ Prefers not to say ❑

If can tell: Date of birth (DD/MM/YY): ________________________________________

If exact date of birth cannot be recalled, what month and year were you born? (MM/YY) ______

**C. Human factors**

Sex: Male ❑ Female ❑

Tribe ___________________________________

Religion: Christian ❑ Moslem ❑ Other ___________________ (please specify)

Highest education level attained: No formal education ❑ Primary ❑ Secondary ❑ Certificate / Diploma ❑ Degree and above ❑

Marital status : Single never married ❑ Single divorced / widowed ❑ Married ❑ Prefer not to say ❑

How many people stay in the household with you? ___________________

Do the people staying in your household include your spouse? Yes ❑ No ❑

Are there teenage children in your home? Yes ❑ No ❑

If yes, how many teenage children in your home? ________________________________

Are you employed? Yes ❑ No ❑ If yes, please specify type of employment ______________________

What is your caretaker’s sex? Male ❑ Female ❑

What is your caretaker’s education level? No formal education ❑ Primary ❑ Secondary ❑ Certificate / Diploma ❑ Degree and above ❑

Are you a Current dog owner? Yes ❑ No ❑

If yes, how many dogs do you own ? _________________________________

If yes, what type / breed of dog? ____________________

What do you use the dog for? _______________________________________________

How many years have you been owning the dog (s)? _________________________

If no, have you ever owned a dog? Yes ❑ No ❑

If yes, where do the dogs stay? Have own house ❑ Out in the compound ❑ Share house with people ❑ Roam around in the village ❑

Do you know of any close relative of yours who own a dog? Yes ❑ No ❑

Have you ever been bitten by a dog before this bite? Yes ❑ No ❑

If yes, in which year did this bite happen ? ___________________________

For this current bite, did you believe before that a dog could attack and bite you ? Yes ❑ No ❑

Were you immunized against rabies prior to being bitten by the dog? Yes ❑ No ❑

If yes, when were you immunized (month and year)? _________________

**D. Dog factors**

What was the sex of the dog? Male ❑ Female ❑ Don’t know ❑

What is the age of the biting dog (months) _______________________

Was the biting dog sick? Yes ❑ No ❑

Was the biting dog exhibiting fear of people? Yes ❑ No ❑

What is the dog used for? Security ❑ Pet ❑ Stray ❑ Dont know ❑

Was the dog vaccinated to the best of your knowledge? Yes ❑ No ❑ Dont know ❑

Was the dog spayed / neutered / castrated? Yes ❑ No ❑ Don’t know ❑

Was the dog on the leash? Yes ❑ No ❑

Has this dog ever bitten someone else before biting you? Yes ❑ No ❑ Don’t know ❑

Did this dog bite another person after biting you? Yes ❑ No ❑ Don’t know ❑

**E. Dog bite circumstances**

**E1: Before the bite**

What day did the dog bite you? (DD/MM/YY) _______________________________

What time of day did the dog bite you? Morning ❑ Evening ❑ Night ❑

At what particular time did the dog bite you ? (12-hour format) ______________________

What it raining when you were attacked by the dog ? Yes ❑ No ❑

If the attack happended at night, was there a visible moon ? Yes ❑ No ❑

Were you bitten by your own dog? Yes ❑ No ❑

If yes, how long had you stayed with the dog before the bite (months) __________

If yes, was this dog borne in your home ? Yes ❑ No ❑

If yes, do you usually allow this dog into the presence of visitors ? Yes ❑ No ❑

Does this dog usually leave the compound unaccompanied? Yes ❑ No ❑

If no, do you know the owner of the dog? Yes ❑ No ❑

If yes, who is the owner ? Neighbor ❑ Person known to me ❑ Community dog ❑

Were you bitten while on the property of the dog owner ? Yes ❑ No ❑

Was the owner around while the dog was attacking you? Yes ❑ No ❑

How would you describe the size of the dog? Small ❑ Medium ❑ Large ❑ Very large ❑

Do you know the breed of the dog ? Yes ❑ No ❑

If yes, what breed was the dog ? ______________________________________

Did you previously know the biting dog? Yes ❑ No ❑

If yes, can you describe the dog? History and type ________________________________________

Did the dog look sick to you? Yes ❑ No ❑

Where were you? Own home ❑ Home of another person known to me ❑ Home of another person not known to me ❑ On the road ❑ Other ❑, please specify ________________________

Were you in company of another person / people? Yes ❑ No ❑

If yes, what is your relationship with these / this person (s)? _______________________________

What were you doing just before the dog bit you? Walking ❑ Seated ❑ Chasing it away ❑ Feeding it ❑ Other ❑, please specify ____________________________

What was the dog doing just before the bite? Please describe __________________________

Did you try to interpret the mood of the dog before the attack? Yes ❑ No ❑

Could you describe to me how you thought the dog's demeanor / mood was just before the bite? __________________

**E2. During the bite**

Did you approach the dog or did it approach you? I approached it ❑ It approached me ❑

Was the dog stationary or moving / mobile? _______________________________

What was the purpose of the interaction with the dog around the time of the bite? ______________

Did you try to fend off the dog as it attacked you? Yes ❑ No ❑

Where did it bite you? Leg ❑ Hand ❑ Arm ❑ Head ❑ Abdomen ❑ Other, please specify __________

How many times did it bite you? One ❑ Two ❑ Three or more ❑

How do you describe the depth of the wounds? Walking ❑ Seated ❑ Chasing it away ❑ Feeding it ❑ Other ❑, please specify ____________________________

Why do you think that the dog bit you? ____________________________________________

What makes you think that? _____________________________________________________

Do you get information about dogs? Yes ❑ No ❑

Where do you get information about dogs from? Friends ❑ Books ❑ School ❑ Family ❑ Other ❑ please specify________________________________________

Do you think that the bite was intentional? Yes ❑ No ❑

Do you think that anybody is to blame for the bite happening? Yes ❑ No ❑

If yes, who? ___________________________________________

**E3. After the bite**

How would you describe the damage the bite did to you? __________________________________

What did you do to the dog after the bite? Chased it away ❑ Killed it ❑ Nothing ❑ It ran away by itself ❑ Other please specify________________________________________

If it was killed, what happened to the carcass ? Decapitated ❑ Buried ❑ Left to rot ❑ I don’t know ❑ Other, please specify _____________________________________

Are you aware the head of the dog had to be taken for examination ? Yes ❑ No ❑

If yes, was the head take for examination? Yes ❑ No ❑

If not own dog, how did the owner react? ______________________________________

How did the owner’s reaction make you feel? _____________________________________

How did the bite affect the rest of your day? ______________________________________

**F. PRACTICES**

Did you do anything to the wound immediately after the bite? Yes ❑ No ❑

If yes, what did you do? Washed with water and soap ❑ Washed with water only ❑ Did not wash ❑ Other, please specify ______________________________

Did you apply anything to the wound immediately after the bite? Yes ❑ No ❑

If yes, what did you apply to the wound? ____________________________________________

Did you think you needed any medical help after the bite? Yes ❑ No ❑

If yes, what did you do? _______________________________________________

Why did you choose to do that? __________________________________________

**G. SES variables**

Could you tell me if you have the following in your house;

| Item | Yes | No |
| --- | --- | --- |
| Radio |  |  |
| Television |  |  |
| Cell-phone |  |  |
| Bicycle |  |  |
| Motorcyle |  |  |
| Motor vehicle |  |  |
| A piece of land |  |  |
| Large farm animals like cattle, goats and sheep |  |  |
| Small farm animals like poultry |  |  |
| A manufactured bed |  |  |

What is the nature of the walls of their house? No bricks ❑ Unburnt bricks ❑ Burnt bricks with mud ❑ Burnt bricks ⁄ stones with cement ❑ Other, please specify ____________________

## PART B: LABORATORY SAMPLE FORM FOR DOG BITE WOUNDS: BACTERIAL ISOLATES AND THEIR ANTIMICROBIAL SUSCEPTIBILITY

**A. Sample details**

- Patient Code / Laboratory Sample Number: _______________________________
- Sample Collection Date: _____________________Sample Collection Time: ________________
- Sample Collector Name: _______________ Sample Collector Phone: _______________
- Health Facility Name: __________________________________________________
- Sampling Point / Area of wound: ________________________________________
- Sample acceptance: Accepted ❑ Rejected ❑
- Reasons for rejection: _________________________________________________

**B. Bacterial growth**

Aerobic: Yes ❑ No ❑ Anaerobic: Yes ❑ No ❑

**C. Bacteria isolated**

| Aerobic bacteria | Anaerobic bacteria |
| --- | --- |
|  |  |
|  |  |
|  |  |
|  |  |

**D. Susceptibility of micro-organisms isolated to antibiotics expressed as either Sensitive or Resistant**

| No. | Bacterial species | Amoxicillin | Metronidazole | Doxycycline | Cotrimoxazole |
| --- | --- | --- | --- | --- | --- |
|  |  |  |  |  |  |
|  |  |  |  |  |  |
|  |  |  |  |  |  |
|  |  |  |  |  |  |
|  |  |  |  |  |  |

Key: S = sensitive, R = resistance, I = Intermediate; zone diameter in standard millimeter units

**E. Additional notes**

______________________________________________________________________________

______________________________________________________________________________

______________________________________________________________________________

______________________________________________________________________________

______________________________________________________________________________

______________________________________________________________________________

______________________________________________________________________________

______________________________________________________________________________

______________________________________________________________________________

The questionnaire used to collect data on factors associated with pre-clinical care practices undertaken for dog bite patients. The variables include patient socio-demographic factors, dog factors and circumstacnes sorrounding the bite event before, during and after it happened.
