# Supplementary material for: The potential for the double risk of rabies and antimicrobial resistance in a high rabies endemic setting: detection of antibiotic resistance in bacterial isolates from infected dog bite wounds in Uganda
Source: Antimicrob Resist Infect Control. 2022 Nov 13;11:142. doi: 10.1186/s13756-022-01181-0 (PMC9655799; doi:10.1186/s13756-022-01181-0)
Supplement: Supplementary file 2 — Additional file 2. Summary of antimicrobial resistance patterns of bacterial pathogens isolated from wound swab cultures among patients with DBWs attending PET centers in Uganda. [file 13756_2022_1181_MOESM2_ESM.docx]

**Additional file 2: Summary of antimicrobial resistance patterns of bacterial pathogens isolated from wound swab cultures among patients with DBWs attending PET centers in Uganda**

| **Bacteria** | **Antimicrobial classes and related number of resistant isolates (%)** | | | | |
| --- | --- | --- | --- | --- | --- |
| **Gram positive** | **Number** | **R_1_** | **R_2_** | **R_3_** | **≥R_4_** |
| *Staphylococcus aureus** | 103 (25.4) | 56 (13.8) | 11 (2.7) | 24 (5.8) | 12 (2.9) |
| *Staphylococcus intermedius** | 30 (7.4) | 2 (6.7) | 15 (3.7) | 13 (3.2) | 0 (0.0) |
| CONS | 68 (16.7) | 53 (13.1) | 15 (3.6) | 0 (0.0) | 0 (0.0) |
| *Streptococuss canis** | 18 (4.4) | 0 (0.0) | 2 (0.5) | 10 (2.5) | 6 (1.5) |
| *Streptococuss pyogenes** | 29 (7.1) | 2 (0.5) | 14 (3.5) | 12 (2.9) | 1 (0.3) |
| Other Streptococci | 12 (2.9) | 11 (2.7) | 1 (0.3) | 0 (0.0) | 0 (0.0) |
| *Bacillus spp** | 11 (2.7) | 2 (0.5) | 3 (0.7) | 4 (1.0) | 2 (0.5) |
| *Enterococcus feacalis** | 19 (4.7) | 4 (1.0) | 9 (2.2) | 2 (0.5) | 4 (1.0) |
| *Enterococcus faecium* | 2 (0.5) | 0 (0.0) | 1 (0.3) | 1 (0.3) | 0 (0.0) |
| Other Enterococci | 6 (1.5) | 5 (1.2) | 1 (0.3) | 0 (0.0) | 0 (0.0) |
| *Micrococcus spp* | 8 (1.9) | 8 (1.9) | 0 (0.0) | 0 (0.0) | 0 (0.0) |
| *Corynebacterium spp* | 33 (8.1) | 18 (4.4) | 9 (2.2) | 6 (1.5) | 0 (0.0) |
| *Gemella morbillorium* | 21 (5.1) | 18 (4.4) | 3 (0.7) | 0 (0.0) | 0 (0.0) |
| *Lactobacillus spp** | 31 (7.6) | 9 (2.2) | 8 (1.9) | 4 (1.0) | 10 (2.5) |
| *Lactococcus spp** | 23 (5.6) | 7 (1.8) | 6 (1.5) | 6 (1.5) | 4 (1.0) |
| **Total** | **406 (100)** | **195 (48.0)** | **98 (24.1)** | **82 (20.2)** | **39 (9.6)** |
| **Gram negative** |  |  |  |  |  |
| *Pasteurella maltocida* | 64 (17.7) | 47 (13.0) | 17 (4.7) | 0 (0.0) | 0 (0.0) |
| *Pasteurella canis* | 26 (7.2) | 19 (5.2) | 7 (1.9) | 0 (0.0) | 0 (0.0) |
| Other pasteurellae | 31 (8.6) | 3 (0.8) | 28 (7.7) | 0 (0.0) | 0 (0.0) |
| *Proteus vulgaris** | 2 (0.6) | 0 (0.0) | 0 (0.0) | 2 (0.6) | 0 (0.0) |
| *Proteus mirabilis** | 7 (1.9) | 2 (0.6) | 1 (0.3) | 2 (0.6) | 2 (0.6) |
| *Pseudomonas aeuroginosa* | 3 (0.8) | 0 (0.0) | 3 (0.8) | 0 (0.0) | 0 (0.0) |
| *Pseudomonas stutzeri* | 3 (0.8) | 3 (0.8) | 0 (0.0) | 0 (0.0) | 0 (0.0) |
| *Pseudomonas alcaligenes* | 2 (0.6) | 0 (0.0) | 0 (0.0) | 0 (0.0) | 2 (0.6) |
| Other pseudomonas* | 11 (3.0) | 4 (1.1) | 3 (0.8) | 2 (0.6) | 2 (0.6) |
| *Klebsiella pneumonae** | 11 (3.0) | 0 (0.0) | 3 (0.8) | 4 (1.1) | 4 (1.1) |
| *Klebsiella oxytoca** | 6 (1.7) | 0 (0.0) | 2 (0.6) | 0 (0.0) | 4 (1.1) |
| *Acinetobacter spp** | 3 (0.8) | 0 (0.0) | 0 (0.0) | 3 (0.8) | 0 (0.0) |
| *Moellerella wisconsensis** | 5 (1.4) | 0 (0.0) | 1 (0.3) | 1 (0.3) | 3 (0.8) |
| *Capnocytophaga canimorsus** | 36 (9.9) | 3 (0.8) | 8 (2.2) | 11 (3.0) | 14 (3.9) |
| *Stenotrophomonas maltophilia* | 4 (1.1) | 3 (0.8) | 1 (0.3) | 0 (0.0) | 0 (0.0) |
| *Bergeyella zoohelcum* | 3 (0.8) | 0 (0.0) | 0 (0.0) | 0 (0.0) | 3 (0.8) |
| *Citrobacter werkmanii** | 1 (0.3) | 0 (0.0) | 0 (0.0) | 1 (0.3) | 0 (0.0) |
| *Citrobacter freundii** | 2 (0.6) | 0 (0.0) | 0 (0.0) | 0 (0.0) | 2 (0.6) |
| *Enterobacter asburiae** | 1 (0.3) | 0 (0.0) | 0 (0.0) | 1 (0.3) | 0 (0.0) |
| Other enterobacter spp | 13 (3.6) | 0 (0.0) | 0 (0.0) | 6 (1.7) | 7 (1.9) |
| *E. coli** | 6 (1.7) | 0 (0.0) | 2 (0.6) | 0 (0.0) | 4 (1.1) |
| *Serratia rubidae* | 2 (0.6) | 0 (0.0) | 2 (0.6) | 0 (0.0) | 0 (0.0) |
| *Serratia entomophila* | 3 (0.8) | 0 (0.0) | 3 (0.8) | 0 (0.0) | 0 (0.0) |
| *Fusobacterium spp* | 48 (13.3) | 40 (11.0) | 8 (2.2) | 0 (0.0) | 0 (0.0) |
| *Bacteriodes spp** | 34 (9.4) | 5 (1.4) | 4 (1.1) | 25 (6.9) | 0 (0.0) |
| *Prevotella spp* | 35 (9.7) | 23 (6.4) | 12 (3.3) | 0 (0.0) | 0 (0.0) |
| **Total** | **362 (100)** | **152 (42.0)** | **105 (29.0)** | **58 (16.0)** | **47 (12.9)** |

Among the 406 gram positive isolates, 121/406 (29.8%) were found to be multidrug resistant. Specifically, these included*, S. intermedius, S. canis,* and *Corynebacterium spp* which were resistant to three classes of antimicrobial agents. In addition, *S. aureus, S. pyogenes, E. feacalis, Lactobacillus spp* and *Lactococcus spp* were resistant to 4 or more classes of antimicrobial drugs.
